# Pitfalls of Ovarian Ablative Magnetic Resonance-guided Radiation Therapy for Refractory Endometriosis

**DOI:** 10.7759/cureus.2294

**Published:** 2018-03-09

**Authors:** Shyama Tetar, Anna Bruynzeel, Omar Bohoudi, Theodoor Nieboer, Frank Lagerwaard

**Affiliations:** 1 Radiation Oncology, VU University Medical Center, Amsterdam, The Netherlands; 2 Department of Gynecology, Radboud University Medical Center

**Keywords:** mrgrt, endometriosis treatment, ovarian ablation, adaptive radiotherapy, adaptive planning

## Abstract

In this case presentation, we describe the challenges of performing magnetic resonance-guided radiation therapy (MRgRT) with plan adaptation in a patient with advanced endometriosis, in whom several prior therapeutic attempts were unsuccessful and extensive pelvic irradiation was regarded as being too toxic. Treatment was delivered in two sessions, first for the seemingly only active right ovary, and at a later stage for the left ovary. Some logistical problems were encountered during the preparation of the first treatment, which were subsequently optimized for the second treatment by using transvaginal ultrasound to determine the optimum time point for simulation and delivery. Using breath-hold gated delivery and plan adaptation, radiation dose to the bowel could be minimized, resulting in good tolerance of treatment. Because of the need to simulate and deliver in a brief optimal time span for visibility of the follicles in the ovaries, a single fraction dose of 8 Gy was used in our patient. Hormonal outcome after her second treatment is still pending.

In conclusion, MRgRT with plan adaptation is feasible for the occasional patient with refractory endometriosis. Simulation and delivery needs to be synchronized with the menstrual cycle, ensuring that the Graafian follicles allow the ovaries to be visible on magnetic resonance imaging (MRI). Because the ovaries are only visible on T2-weighted MRI for a very brief period of time, we suggest that it is preferable to use single fraction radiotherapy with a brief interval between simulation imaging and delivery.

## Introduction

Endometriosis constitutes a benign gynecological condition that is characterized by the presence of ectopic endometrial-like tissues. Endometriosis is a chronic inflammatory disorder that can significantly impact upon the quality of life with symptoms ranging from dysmenorrhea to pelvic or abdominal bowel symptoms, and in severe cases even obstruction or perforation [1]. Although pain medication, hormonal therapy, or surgery form the mainstay of treatment for symptomatic patients, radiotherapy, usually in the form of extensive pelvic irradiation, has been applied in exceptional cases with refractory disease [2-3]. In this case presentation, we describe the challenges of performing magnetic resonance-guided radiation therapy (MRgRT) in a patient with advanced endometriosis, in whom several prior therapeutic attempts were unsuccessful and extensive pelvic irradiation was regarded as being too toxic.

## Case presentation

A 44-year-old woman with an extensive history of advanced and refractory endometriosis presented with abdominal complaints associated with a pseudo-obstruction bowel syndrome. She had previously undergone multiple abdominal surgeries, including a subtotal colectomy. She did not respond to ovarian suppression with gonadotropin-releasing hormone agonist, and a recent embolization of both ovarian arteries only achieved a partial and temporal hormonal response. Repeated post-embolization transvaginal ultrasound investigations could only discriminate the right ovary; the atrophic left ovary could not be visualized. Surgical ovariectomy was considered to be high-risk because of extensive abdominal and pelvic adhesions on a diagnostic magnetic resonance imaging (MRI). She subsequently was referred by her treating gynecologist for radiation to the right ovary in order to achieve a postmenopausal hormonal status. Because the patient suffered from chronic and severe bowel complaints with a need for total parenteral nutrition, extensive pelvis radiation, a historical method for ovarian ablation, was considered contraindicated. MR-guided radiotherapy, a recently introduced advanced technique, was preferred in order to optimally spare the surrounding bowel and selectively treat the right ovary.

**Figure 1:**
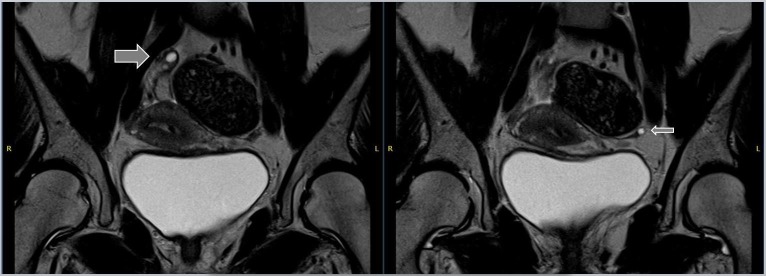
Coronal images from the diagnostic magnetic resonance imaging (MRI) scan Coronal T2-weighted images from the diagnostic MR scan showing the right ovary with three small follicles (thick arrow) with a maximum diameter of 15 mm. The left ovary is not clearly recognizable, but a small cyst is visible with a maximum diameter of 8 mm at the position of the ovary (thin arrow).

In preparation of MRgRT, on the day of the initial consultation, a simulation (SIM) MR scan was performed on MRIdian (Viewray Inc., Mountain View, US). The SIM MR scan was acquired in shallow breath-hold in order to restrict respiratory artifacts. The MRIdian uses a 0.35 Tesla magnetic field with a balanced steady state free precession (bSSFP) sequence. In this sequence on the MRIdian, there is heavy T2 weighting, hence the fluid and the ovarian follicles are bright. However, as this acquired SIM MR scan did not take the phase in the menstrual cycle into account, none of the ovaries were clearly detectable. This was confirmed by an experienced radiologist specialized in abdominal MRI who advised to perform a diagnostic MR scan first. This scan was performed three weeks later and showed multiple follicles in the right ovary, with only a small cyst at the location of the left ovary (Figure 1).

**Figure 2:**
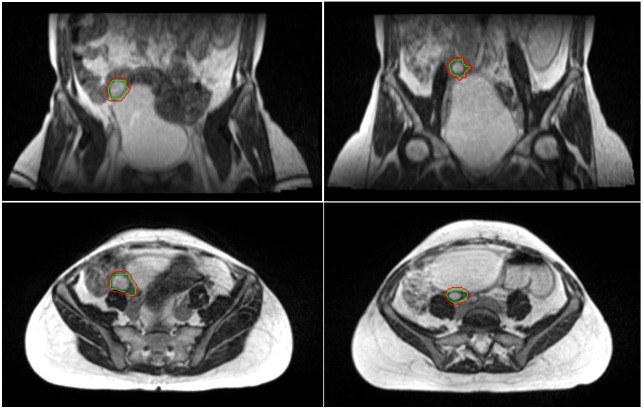
Simulation scan versus pretreatment scan A coronal and axial view of the simulation scans is displayed on the left panels. On the right, a coronal and axial view of radiotherapy treatment fraction is seen. Green = the right ovary. Red = treatment boundary.

With the duration of the menstrual cycle in mind, the SIM MR scan was repeated one month later showing a comparable image of the ovaries as on the diagnostic MRI. This SIM MR was used for the generation of a baseline plan, prescribing 8 Gy in a single fraction to the right ovary. Several days after the SIM MR, the patient returned for the scheduled treatment. Unfortunately, because she appeared to be in a postovulatory phase of her menstrual cycle, the right ovary could not be seen on the pretreatment MR scan. Two more attempts in the weeks thereafter had to be performed before the right ovary was visible, and treatment could actually be delivered. Plan re-optimization was useful, because both the position and volume of the right ovary were different on the SIM MR and the pretreatment MR (Figure 2). Treatment was delivered in shallow inspiration breath-hold with a 3 mm gating boundary between the right ovary and the planning target volume (PTV).

The patient reported mild symptoms of an inflated belly and some fatigue for which no intervention was required in the first days following treatment, both scored as grade 1 toxicity (CTCAE criteria v. 4.3). She experienced neither nausea nor diarrhea. Hormonal assessment at four months follow-up revealed a serum estradiol (E2) of 40 pmol/L and serum follicle stimulating hormone (FSH) level of 70 U/L, indicating that she biochemically had reached a postmenopausal status. In addition to the hot flushes she experienced, the patient also reported having fewer abdominal complaints. Unfortunately, at six months follow-up, the patient appeared to have regained a biochemical premenopausal status. In addition, this was confirmed by a transvaginal ultrasound that showed follicles, this time in an apparently active left ovary. As a result of this finding, the patient was referred for local treatment by radiotherapy, now for her left ovary.

Having learned from the first procedure, this time the MRgRT was synchronized with her menstrual cycle by performing weekly transvaginal ultrasound in order to determine the appropriate treatment window. As soon as the follicle was visible in the left ovary, the patient was seen at our department with immediate MR simulation. After quick planning, the patient was treated within 24 hours of the SIM MR scan with a single dose of 8 Gy. Similar to the first treatment, the patient noticed mild abdominal complaints and fatigue in the first days following the second treatment. Toxicity did not impact upon her daily activities and was therefore scored as grade 1. At one month after treatment, she reported that her bowel symptoms were better than in previous years. She had even been able to discontinue total parenteral nutrition. The biochemical results after radiotherapy on the left ovary are pending.

## Discussion

This case report describes an uncommon indication for radiotherapy with the goal to obtain ovarian ablation in a patient with refractory and advanced endometriosis. Given the extensive bowel complications in this patient, neither surgical ovariectomy nor extensive pelvic radiation was considered feasible. It was contemplated that MR-guided focal radiotherapy directed towards the remaining active right ovary might be beneficial in order to diminish potential gastro-intestinal side effects. One main difference between conventional radiotherapy (such as e.g., extensive pelvic irradiation) and MRgRT is that setup and radiation delivery is delivered to a specific target rather than a region. Contrary to conventional radiotherapy, this necessitates that the ovary is discernible on SIM and pretreatment MR scans.

Diagnostic MR imaging of the normal ovaries is usually performed using T2-weighted imaging, which makes the bSSFP sequence of the MRIdian suitable for visualizing the ovaries. During the menstrual cycle, the Graafian follicles can be seen as bright cysts within the ovarian stroma, and this pre-ovulatory phase is ideal for imaging. Both simulation and MR-guided delivery, therefore, have to take place in the last days of this relatively short time slot. During the first treatment fraction, we used ‘trial and error’ and the irregular cycle of our patient necessitated several visits. In anticipation of the second treatment, simulation and delivery were synchronized with her menstrual cycle by performing sequential transvaginal ultrasound investigations. Once the follicles were visible on this imaging, simulation and delivery took place within 24 hours. For the same reason, we used single fraction radiotherapy, rather than a more prolonged fractionation scheme. Clinical experience of ovarian ablative radiotherapy is mostly derived from older literature in breast cancer patients and has only been reported in case reports for refractory endometriosis [2-3]. While radiotherapy for endometriosis is most commonly delivered in the form of extensive pelvic irradiation in 15-20 Gy in 10 fractions, the literature on hormonal castration in breast cancer patients describes a wider range of fractionation schemes, including the use of single-fraction irradiation. Because the most frequently applied radiation scheme in the literature is 15 Gy in 5 fractions [4], we selected a single fraction dose of 8 Gy which has a comparable biological equivalent dose for an α/β of 3 and slightly lower than that for an α/β of 10.

**Figure 3:**
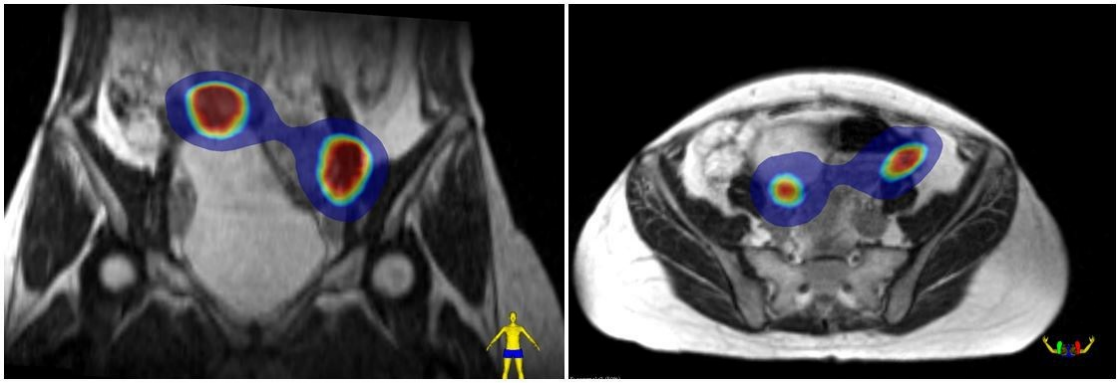
The cumulative dose distribution A coronal and axial view of dose accumulation of both treatments projected on the first simulation magnetic resonance (SIM MR) scan. Blue color wash = 4Gy. Inner red color wash = 8 Gy.

From a practical standpoint, we performed a scan during the shallow breath-hold and with a full bladder to optimize visibility of the ovary, minimize target (and bowel) mobility, and enable bowel spacing. Plan adaptation was used to generate a conformal as possible dose distribution. The cumulative dose distribution from both fractions can be seen in Figure 3, which clearly illustrates the resulting relative sparing of the surrounding bowel, especially when compared with conventional extensive pelvis irradiation. Despite two treatments with 8 Gy, the acute toxicity has been minimal with temporary bowel symptoms and some fatigue. The results of hormonal outcome in our patient are pending.

## Conclusions

MRgRT with plan adaptation is feasible for the occasional patient with refractory endometriosis. Simulation and delivery needs to be synchronized with the menstrual cycle, ensuring that the Graafian follicles allow the ovaries to be visible on MRI. Because the ovaries are only visible on T2-weighted MRI for a very brief period of time, we suggest that it is preferable to use single fraction radiotherapy with a brief interval between simulation imaging and delivery.
